# State-dependent filtering as a mechanism toward visual robustness

**DOI:** 10.3389/fncom.2025.1699179

**Published:** 2025-12-10

**Authors:** Jing Yan, Yunxuan Feng, Wei P. Dai, Yaoyu Zhang

**Affiliations:** 1 School of Mathematical Sciences, Institute of Natural Sciences and MOE-LSC, Shanghai Jiao Tong University, Shanghai, China; 2 Research Institute of Intelligent Complex Systems, Fudan University, Shanghai, China; 3 Shanghai Artificial Intelligence Laboratory, Shanghai, China

**Keywords:** ring model, robustness, lateral connections, state-dependent filtering, visual processing

## Abstract

Robustness, defined as a system's ability to maintain functional reliability in the face of perturbations, is achieved through its capacity to filter external disturbances using internal priors encoded in its structure and states. While biophysical neural networks are widely recognized for their robustness, the precise mechanisms underlying this resilience remain poorly understood. In this study, we explore how orientation-selective neurons arranged in a one-dimensional ring network respond to perturbations, with the aim of uncovering insights into the robustness of visual subsystems in the brain. By analyzing the steady-state dynamics of a rate-based network, we characterize how the activation state of neurons influences the network's response to disturbances. Our results demonstrate that the activation state of neurons, rather than their firing rates alone, governs the network's sensitivity to perturbations. We further show that lateral connectivity modulates this effect by shaping the response profile across spatial frequency components. These findings suggest a state-dependent filtering mechanism that contributes to the robustness of visual circuits, offering theoretical insight into how different components of perturbations are selectively modulated within the network.

## 1 Introduction

Robustness—the ability of a system to maintain its functionality in the face of perturbations—is a critical property of many complex systems, including neural networks (Kitano, 2004; Alderson and Doyle, 2010). The visual system, in particular, exhibits remarkable robustness, accurately perceiving and recognizing objects despite variations in lighting, viewpoints, and other distortions in the visual scene (DiCarlo et al., 2012). Understanding the mechanisms that enable such stability is a central goal in computational neuroscience and has direct implications for the development of reliable artificial visual systems. This raises a fundamental question: How does robustness develop from the architecture and dynamics of cortical circuits?

A growing body of research suggests that cortical computations are dynamically shaped by internal states and recurrent interactions. Normalization provides a canonical account of gain control and robustness (Carandini and Heeger, 2012), predictive–processing frameworks emphasize adaptive feedback and contextual modulation (Keller and Mrsic-Flogel, 2018; Mante et al., 2013), and circuit-level studies link connectivity structure to emergent, state-dependent computations (Mastrogiuseppe and Ostojic, 2018; Stringer et al., 2019; Rubin et al., 2015). Building on these advances, we focus on the specific contribution of lateral connectivity to robustness, independent of higher-area feedback. Our contribution is to provide a compact, quantitative framework that formalizes state-dependent filtering by analyzing the system's Jacobian through singular value decomposition (SVD) as an analytical tool for characterizing sensitivity. This analysis identifies maximally amplified perturbation modes and demonstrates how they correspond to perceptually interpretable transformations—such as contrast modulation, small rotations, and elongation—via a Gabor mapping. Together, these results clarify how lateral connectivity and activation state jointly implement selective robustness within a simplified cortical model.

To isolate the role of lateral connectivity, simplified recurrent architectures such as the ring model have proven particularly useful. The ring model, consisting of orientation-selective neurons with Gaussian-shaped connectivity on a one-dimensional ring, has been widely used to study fundamental aspects of visual processing, including orientation selectivity (Ben-Yishai et al., 1995), contrast invariance (Carandini and Heeger, 2012), surround suppression (Somers et al., 1995; Sompolinsky and Shapley, 1997; Rubin et al., 2015), and binocular rivalry and fusion (Said and Heeger, 2013; Wilson, 2017; Wang et al., 2020). Despite its simplicity, the ring model can incorporate effective single-neuron nonlinearities and experimentally derived connectivity profiles, enabling tractable analysis of how neuronal states and recurrent interactions shape network responses.

In this study, we investigate the state-dependent response of the ring model to structured perturbations and its implications for visual robustness. We first analyze a steady-state rate-based ring model and derive analytical expressions relating perturbation responses to activation states and connectivity. We then identify the perturbations that elicit the largest state-dependent responses and examine their functional properties. To validate these results, we extend the analysis to a more biologically plausible spiking version of the ring model. Finally, by mapping the network's orientation-domain responses into image space through Gabor filters, we demonstrate that structured perturbations—such as contrast or aspect-ratio modulation—induce far stronger responses than random noise. By elucidating the model's state-dependent responses to perturbations, we instantiate the priors that filter external perturbations and pave the way for developing more robust and biologically inspired artificial vision systems.

## 2 Results

### 2.1 Robustness as selective filtering

In this study, we define robustness not merely as insensitivity to external noise, but as the capacity to selectively filter perturbations based on their semantic relevance—that is, the extent to which a perturbation modifies perceptually or behaviorally meaningful aspects of the input.

As discussed in (Goodfellow et al. 2014), small adversarial perturbations—imperceptible to human observers—can cause deep neural networks to produce drastically incorrect outputs. For example, a slight input modification may cause a model to misclassify a panda as a gibbon, even though there is no significant semantic change in the image. In this case, the system is not robust because it reacts disproportionately to irrelevant noise.

**Figure 1:**
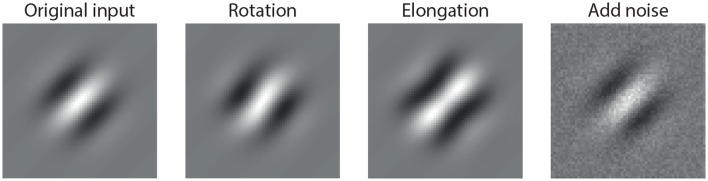
Example of perturbations applied to a standard Gabor stimulus: (1) original input, (2) rotated version (orientation change), (3) elongated version (spatial shape change), and (4) noise-added version (random perturbation). While all three perturbations are small in magnitude, only the first two induce semantically meaningful changes in the stimulus, which a robust visual system should prioritize. The last perturbation, although visually subtle, is semantically irrelevant and ideally should be suppressed.

In contrast, a biologically inspired visual system should instead prioritize perturbations that correspond to meaningful changes in the input—such as modifications in shape, orientation, or contrast—while suppressing perturbations that do not alter perceptual semantics. These types of perturbations are illustrated in Figure 1, where we show example modifications to a Gabor stimulus that are perceptually salient yet small in magnitude.

This perspective motivates our study: we hypothesize that robustness in visual processing relies on an internal filtering mechanism that adapts based on the input. In particular, the model should exhibit state-dependent filtering, in which the response to a perturbation depends on the current activation pattern induced by the input.

To explore whether biologically inspired systems could support such selective filtering, we study a simplified model of the primary visual cortex—the ring model. We provide a method to compute the perturbation direction that maximizes the change in the model response across different activation patterns. Remarkably, when these perturbations are mapped back into the image domain, they often resemble structured patterns such as rotation, elongation, or contrast change. In contrast, random noise-like perturbations of the same energy are consistently attenuated by the model, indicating that the system selectively filters out semantically irrelevant inputs.

This finding supports the hypothesis that robust computation may emerge from state-dependent filtering, in which the structure of meaningful perturbations aligns with the system's internal sensitivity.

Formally, the sensitivity of the ring model to small perturbations can be described by the Jacobian operator:


$$\begin{array}{l} DF\left({\text{r}}_{0}\right):=\frac{\partial \text{r}}{\partial \text{I}}{|}_{\text{r}={\text{r}}_{0}}, \end{array}$$ (1)

which maps infinitesimal changes in input **Δ*I*** to changes in system response **Δ*r*** around a fixed operating point **r**_0_. Applying singular value decomposition (SVD) to *DF*(**r**_0_) yields orthogonal perturbation directions, called singular vectors, each associated with an amplification factor, the corresponding singular value. The right singular vectors indicate orthogonal directions in the input space that produce maximal response changes, while the singular values quantify the corresponding frequency response magnitude. Intuitively, singular vectors represent the most “effective perturbations” of the system: those aligned with large singular values cause strong changes in network activity, whereas those aligned with small singular values are effectively filtered out. This provides a principled mathematical framework linking our semantic notion of robustness to structured perturbation patterns, such as orientation shifts or elongation, that we will analyze in subsequent sections.

### 2.2 The perturbed system: determined by lateral connections and activation pattern

The ring model provides a simplified framework for studying orientation columns in the primary visual cortex (V1), where neurons' preferred orientations are arranged on a ring. It contains excitatory (E) and inhibitory (I) populations, connected through Gaussian-shaped kernels. We begin our analysis with a steady-state rate-based version of the ring model (see Section 4), defined by the following system of equations:


$$\begin{array}{l} r_{E}=g\left(I_{E}+k_{EE}*r_{E}-k_{EI}*r_{I}\right),\\ r_{I}=g\left(I_{I}+k_{IE}*r_{E}-k_{II}*r_{I}\right), \end{array}$$ (2)

where *r*_*X*_ denotes the firing rate vector of population *X* ∈ {*E, I*}, *I*_*X*_ the external input, and *k*_*XY*_ the lateral connectivity kernel from population *Y* to *X*, where *Y* ∈ {*E, I*} as well. The convolution operation (*) is circular, enforcing the ring topology over preferred orientations (see Section 4). The activation function *g* is ReLU, chosen for its biological plausibility and analytical tractability.

Linearizing around a fixed operating point results in the perturbed system


$$\begin{array}{l} \delta r_{E}=g_{E}^{\prime }⊙\left(\delta I_{E}+k_{EE}*\delta r_{E}-k_{EI}*\delta r_{I}\right),\\ \delta r_{I}=g_{I}^{\prime }⊙\left(\delta I_{I}+k_{IE}*\delta r_{E}-k_{II}*\delta r_{I}\right). \end{array}$$ (3)

where $g_{X}^{\prime }$ is binary (0 or 1), depending on whether the neuron is active, and ⊙ denotes element-wise multiplication. In matrix form, with diagonal matrices $G_{X}=\text{diag}\left(g_{X}^{\prime }\right)$ and circulant matrices *K*_*XY*_, (see Section 4): Equation 2


$$\begin{array}{l} \delta r_{E}=G_{E}\left(\delta I_{E}+K_{EE}\delta r_{E}-K_{EI}\delta r_{I}\right),\\ \delta r_{I}=G_{I}\left(\delta I_{I}+K_{IE}\delta r_{E}-K_{II}\delta r_{I}\right), \end{array}$$ (4)

The formal solution is as follows:


$$\begin{array}{l} \delta r_{E}={\left(I-G_{E}K_{EE}+G_{E}K_{EI}{\left(I+G_{I}K_{II}\right)}^{-1}G_{I}K_{IE}\right)}^{-1}\\ \;\left(G_{E}\delta I_{E}-G_{E}K_{EI}{\left(I+G_{I}K_{II}\right)}^{-1}G_{I}\delta I_{I}\right),\\ \delta r_{I}={\left(I+G_{I}K_{II}\right)}^{-1}G_{I}\left(\delta I_{I}+K_{IE}\delta r_{E}\right), \end{array}$$ (5)

This solution reveals how perturbations to the input propagate through the network, depending on both the active set of neurons and the structure of lateral connectivity. Since *G*_*X*_ encodes the current activation pattern and *K*_*XY*_ the connection topology, the system effectively implements a state-dependent linear filter. Different activation states modulate the frequency response function and the direction of input perturbations, thereby enabling the network to selectively amplify structured perturbations aligned with the activation state while suppressing irrelevant ones.

In the following sections, we analyze this linearized system from multiple perspectives to uncover how state-dependent filtering emerges and contributes to robust computation.

#### 2.2.1 Effects of lateral connections on model response with fully active neurons

We first consider the case that all neurons are active. In this case, the perturbed system can be solved in spatial frequency space, implying that sinusoids are eigenvectors: they retain their shape but only change in intensity as they pass through the system. The change in intensity, which we call frequency response from now on, is determined by the lateral connections. The specific relation is given by the following equation:


$$\begin{array}{l} {\hat{\delta r}}_{E}={\left(1-{\hat{k}}_{EE}+\frac{{\hat{k}}_{EI}{\hat{k}}_{IE}}{1+{\hat{k}}_{II}}\right)}^{-1}\left({\hat{\delta I}}_{E}-\frac{{\hat{k}}_{EI}}{1+{\hat{k}}_{II}}{\hat{\delta I}}_{I}\right), \end{array}$$ (6)

where $\hat{v}$ denotes the DFT (Discrete Fourier Transform) of a vector *v*. Details are shown in Section 4. Here, we have suppressed the element-wise multiplication symbol (⊙) for notational simplicity, as all multiplications in frequency space are understood to be element-wise. We focus on the excitatory population since it provides the main output of the ring model and is most relevant for the downstream readout, and we will keep this focus throughout the following analyses. We denote


$$\begin{array}{l} {ĥ}_{-1}:={ĥ}_{0}^{-1}:={\left(1-{\hat{k}}_{EE}+\frac{{\hat{k}}_{EI}{\hat{k}}_{IE}}{1+{\hat{k}}_{II}}\right)}^{-1}, \end{array}$$ (7)

which we call the frequency response function from now on.

We assume that inhibitory-to-inhibitory (I–I) connections are absent, i.e., ${\hat{k}}_{II}=0$, for analytical simplicity and interpretability. Although such connections exist in biological circuits and may contribute to overall inhibitory modulation, they are not essential for the frequency-selective filtering we focus on. Omitting them allows us to highlight the balance between excitation and inhibition in shaping the model's robustness.

Lateral connections can be divided into two parts: the excitatory term ${\hat{k}}_{EE}$, which enhances signals through recurrent excitation, and the recurrent inhibitory term ${\hat{k}}_{EI}{\hat{k}}_{IE}$, which reduces signals through the E → I → E pathway. Assuming Gaussian kernels,


$$\begin{array}{l} k_{XY}\left(x\right)=\alpha_{XY}\exp\left(-\frac{x^{2}}{2\sigma_{XY}^{2}}\right),\;X,Y\in \left\{E,I\right\}, \end{array}$$ (8)

where α_*XY*_ sets the connection strength and σ_*XY*_ the spatial spread.

Because Gaussian kernels remain Gaussian in frequency space, both excitation and inhibition act as low-pass filters: excitation boosts low frequencies, while recurrent inhibition suppresses them. High-frequency perturbations, in contrast, pass almost unchanged. The range of affected frequencies scales inversely with σ_*XY*_: broader connections suppress or enhance lower frequencies. Since *k*_*IE*_ and *k*_*EE*_ often share similar widths, the inhibitory term (*k*_EI_*k_*IE*_) typically spans a broader frequency range, reducing lower frequencies more strongly than excitation enhances them.

**Figure 2:**
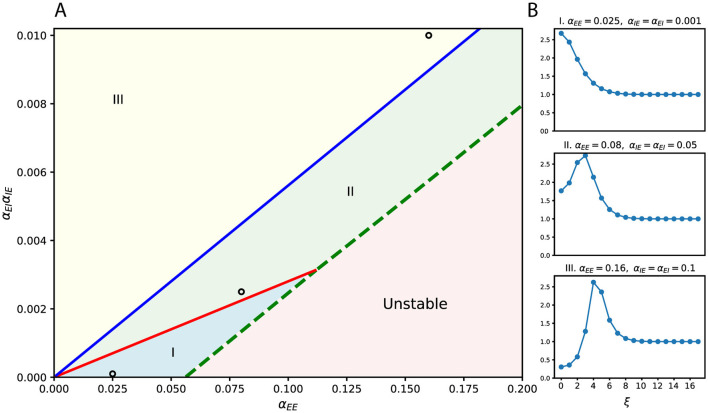
Frequency-selective effects of lateral connectivity. **(A)** Phase diagram over connection strengths, with α_*EE*_ on the horizontal axis and α_*EI*_α_*IE*_ on the vertical axis. The diagram is divided into three stable regions (I–III) and one unstable region, separated by critical lines. Example parameter choices are marked by circles. **(B)** Frequency (ξ) response curves corresponding to the marked parameters in **(A)**. Different regions of the phase diagram produce qualitatively distinct filtering profiles. Here, the widths of lateral connections are fixed and equal (σ_*EE*_ = σ_*EI*_ = σ_*IE*_ = 10).

The resulting frequency-dependent amplification profiles can be summarized in a phase diagram (Figure 2A), parameterized by the widths σ_*EE*_, σ_*EI*_, σ_*IE*_. Distinct regions of stability (I, II, and III) and instability are determined by the ratios α_*EE*_ and α_*EI*_/α_*IE*_. Each stable region corresponds to a characteristic shape of the frequency response curve (Figure 2B), where we plot only the first several dominant frequencies associated with the largest singular values. These already capture the main trend of frequency selectivity. As parameters vary or as the system transitions between regions, the frequency selectivity shifts accordingly.

In short, lateral connectivity controls which perturbation frequencies are amplified or suppressed, acting as a tunable filter that balances excitation and inhibition. This frequency-selective mechanism lays the foundation for robustness: it boosts structured perturbations while suppressing noise-like ones. In later sections, we will further show that these frequency preferences correspond to concrete image-level patterns, such as rotation or elongation.

#### 2.2.2 Effect of activation patterns on model response

We next discuss how the system's behavior changes with different activation patterns, focusing on perturbations that can lead to strong responses.

For a fixed activation pattern *G* = (*G*_*E*_, *G*_*I*_), the perturbed system remains linear. We therefore analyze the excitatory population's sensitivity using the singular value decomposition (SVD) of the corresponding linear operator. Singular vectors of this operator identify perturbations that are maximally amplified; their singular values *s* quantify the amplification.

**Figure 3:**
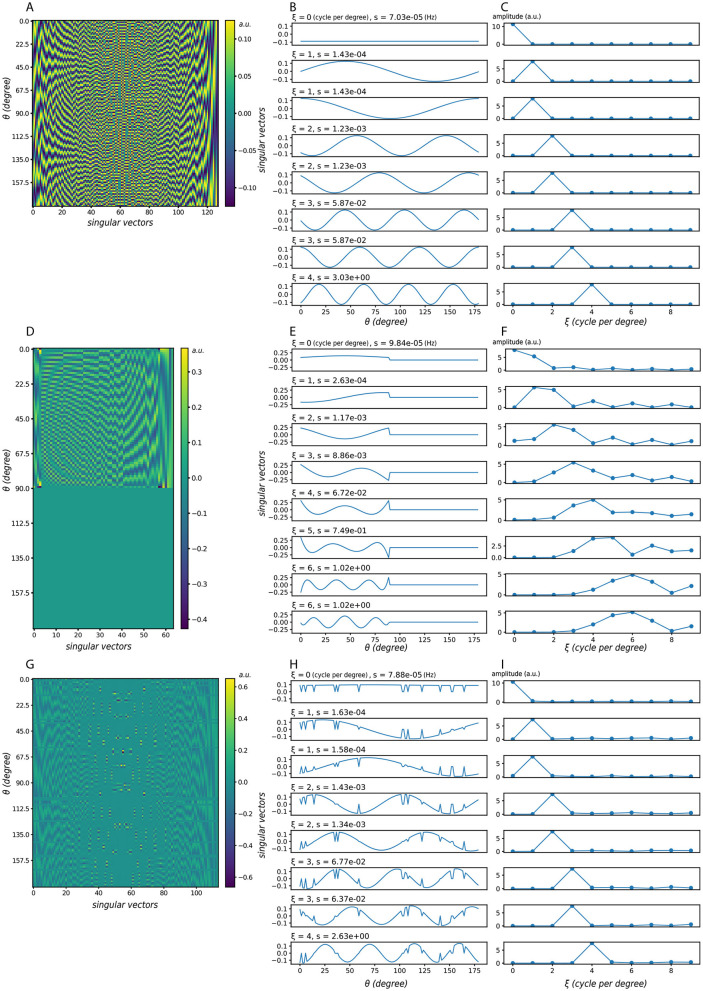
Singular vectors under different activation patterns. **(A–C)** All neurons are active. **(D–F)** Half of the neurons are active in succession. **(G–I)** Random neuron loss, where each neuron is silenced independently with probability 0.1. In each case: **(left)** singular vector matrices, where each column corresponds to one singular vector, ordered by decreasing singular value *s*; **(middle)** the same singular vectors plotted in orientation space, but reordered according to their dominant frequency ξ; **(right)** corresponding frequency-domain representations, where the peak indicates the dominant frequency and its height reflects the strength of that component. Here ξ denotes the dominant frequency of each singular vector, and *s* the corresponding singular value (amplification factor). Despite partial deactivation, the dominant frequency components remain visible, showing that the system's preference for frequency-dominant perturbations is robust to neuron loss. Model parameters: σ_*EE*_ = σ_*EI*_ = σ_*IE*_ = 24, α_*EE*_ = 4, α_*EI*_ = α_*IE*_ = 2.

Using Gaussian-shaped lateral connections, we compute the singular vectors under three representative activation conditions (Figure 3). Figures 3A–C show the case with all neurons active; Figures 3D–F show a case where half of the neurons are consecutively active; Figures 3G–I introduce random neuron loss, where each neuron is independently silenced with probability 0.1. Across all conditions, the singular vectors in orientation space are sinusoidal or resemble sinusoidal patterns. When the same vectors are reordered by their dominant frequency ξ (middle column) and plotted in the frequency domain (right column), their spectra exhibit clear peaks, indicating frequency-dominant structure.

**Figure 4:**
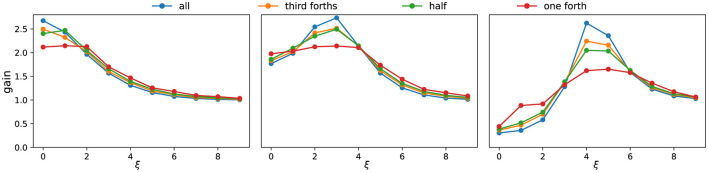
Frequency response curves under different activation patterns. Each subpanel corresponds to one parameter setting (same as Figure 2B). Colors indicate different fractions of consecutively active neurons. The *x*-axis denotes the dominant frequency ξ of the singular vectors, while the *y*-axis shows the corresponding singular value *s*. As the proportion of active neurons decreases, the frequency response curve becomes smoother, reflecting weaker frequency selectivity.

These observations allow us to summarize the system's selectivity using frequency response curves (Figure 4). As the active set shrinks, the gain profile becomes smoother and less sharply tuned: peaks broaden, and their energy redistributes into neighboring frequencies. Peak locations are largely stable across conditions; when shifts occur, they are slight and infrequent. For clarity, Figure 4 displays only the first few dominant frequencies, which already capture the main trend. In terms of magnitudes, the largest singular values *s* associated with the most selective components often decrease modestly as activation is reduced, whereas nearby components can remain comparable or increase, reflecting the redistribution of energy across adjacent frequencies.

In summary, activation patterns strongly shape the system's filtering properties. By changing which frequencies are emphasized, they determine the orientation and directional preferences of the effective filter. Robustness thus arises from this state-dependent filtering: even when only part of the population is active, the network continues to favor structured, frequency-dominant perturbations.

### 2.3 Results for spiking ring models

To validate the generality of our theoretical findings, we conducted experiments using a more biologically realistic conductance-based spiking neuron model. Although the steady-state rate model and the spiking neuron model differ significantly in their implementations, important terms such as 'firing rates' and 'lateral connections' are preserved across both models (see Section 4).

Based on previous study with this model, we consider the mean-driven regime (Cai et al., 2004). The mean-driven regime is more compatible with our theoretical analysis while still capturing biological characteristics. We obtained results in the mean-driven regime that fully align with the steady-state rate model.

**Figure 5:**
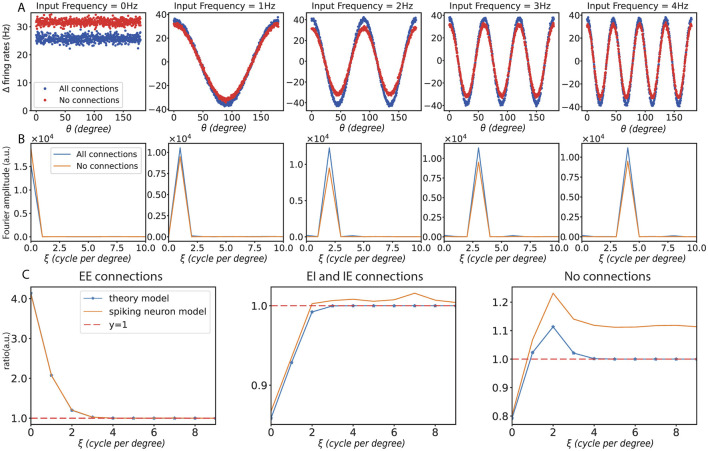
Results with spiking neuron model. **(A)** The changes in firing rates caused by different input frequencies under the condition of full connectivity. **(B)** The corresponding changes in firing rates are shown in frequency space under the same conditions. **(C)** Frequency curves under different connection conditions: the left panel shows the frequency response curve with only recurrent excitation (EE) connections, the middle panel shows the curve with both recurrent excitation (EI) and inhibition (IE) connections, and the right panel shows the frequency response curve without any connections. In all subfigures, ξ represents input frequency.

First, we demonstrate the response of different frequency perturbations in orientation space and frequency space for unconnected and fully connected networks, as shown in Figures 5A, B. Then, we present experimental results in the mean-driven regime. Consistent with the steady-state rate model, the spiking neuron model in the mean-driven regime also demonstrates a preference for different frequencies. This quantitative consistency could be achieved by considering a dimensionful mapping from the dimensionless steady-state rate model to the dimensionful spiking neuron model. As shown in Figure 5C, when no connections were present, the ring model responded uniformly to all frequencies. With only recurrent excitatory connections, the ring model enhanced the low-frequency response. In contrast, when only recurrent inhibitory connections were present, the ring model suppressed the low-frequency response. And when recurrent excitatory and inhibitory connections were present, the model demonstrated a preference for a specific frequency.

### 2.4 From orientation-domain frequencies to image-space perturbations

#### 2.4.1 Gabor operator modes: full vs. partial activation

**Figure 6:**
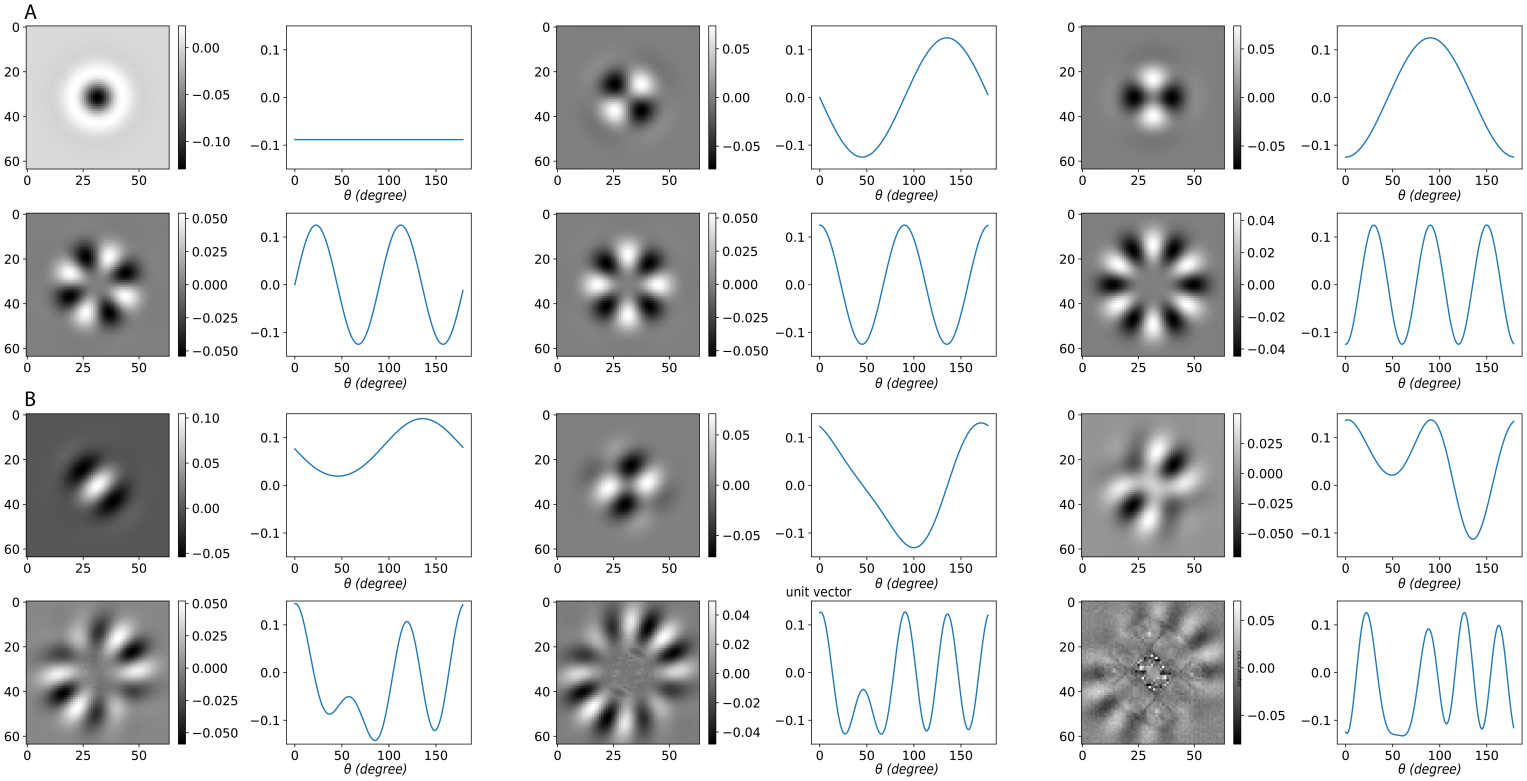
SVD of the Gabor operator under different activation conditions. **(A)** Fully active case. Because each orientation channel is a rotated copy of the same kernel, the Gabor operator $F_{G}$ is approximately block-circulant along the orientation. Its left singular vectors *U* (orientation domain) align with discrete Fourier modes (sinusoids over orientation), shown as waveforms, while the paired right singular vectors *V* (image domain) are reshaped into grayscale image patterns. The *x*-axis of these waveforms denotes the orientation angle θ, and the *y*-axis indicates the relative weight of each singular vector across orientation channels. In this symmetric setting, no directional bias appears; the organizing index is the orientation frequency ξ. **(B)** Partially active case with a contiguous active sector. Restricting $F_{G}$ to a subset of active orientations breaks circular symmetry and introduces directional preference. The leading singular vectors remain frequency-dominant but become biased toward the active sector. Low-order frequencies correspond to familiar image-level perturbations: ξ = 0 represents contrast modulation; ξ = 1 corresponds to a small rotation (odd, shift-like); and ξ = 2 corresponds to elongation or aspect-ratio change (even, two-lobe). Each image tile shows the right singular vector *V* (image-space pattern), with the paired left singular vector *U* plotted as a sinusoid over orientation. Here, ξ denotes the dominant orientation frequency of the singular vector (DFT index).

To link orientation-domain analysis with image space, we examined the singular value decomposition (SVD) of the Gabor operator $F_{G}$, which maps an image to orientation responses (see Section 4). With all orientations active, $F_{G}$ is approximately block-circulant along the orientation dimension. Its singular vectors therefore align with discrete Fourier modes: the orientation-domain vectors are sinusoids, and their paired image-domain vectors are structured stripe patterns (Figure 6A). These modes are arranged from left to right and top to bottom in order of decreasing singular value *s*. Due to the circular symmetry in this fully active case, no directional preference emerges; the organizing index is solely the orientation frequency ξ.

When $F_{G}$ is restricted to a contiguous subset of active orientations, producing a sub-matrix ${\tilde{F}}_{G}$, the circular symmetry is broken. In this scenario, we follow the idea underlying singular value decomposition: we use an optimization procedure to extract patterns in the image space that are mutually orthogonal and satisfy the constraint


$$||{\tilde{F}}_{G}^{c}p{||}_{\infty }\leq ||{\tilde{F}}_{G}p{||}_{\infty },$$

where *p* denotes a candidate pattern. The goal is to identify those patterns that retain the most “energy,” meaning that they produce outputs with the largest norm under these constraints. Although this is not a literal SVD, the construction parallels its logic, so we continue to refer to the resulting modes as singular vectors for consistency. As Figure 6B shows, the leading singular vectors remain frequency-dominant but are biased toward the active sector. In this setting, low-order frequencies correspond to interpretable image-level perturbations: ξ = 0 corresponds to contrast modulation, ξ = 1 to small rotations of oriented content, and ξ = 2 to elongation or aspect-ratio changes. These patterns are also arranged in decreasing order of singular value. This parallel between Gabor SVD modes and ring-model frequency modes shows that both systems naturally produce frequency-dominant eigenmodes that can be aligned.

#### 2.4.2 Perturbation analysis with Gabor inputs

**Figure 7:**
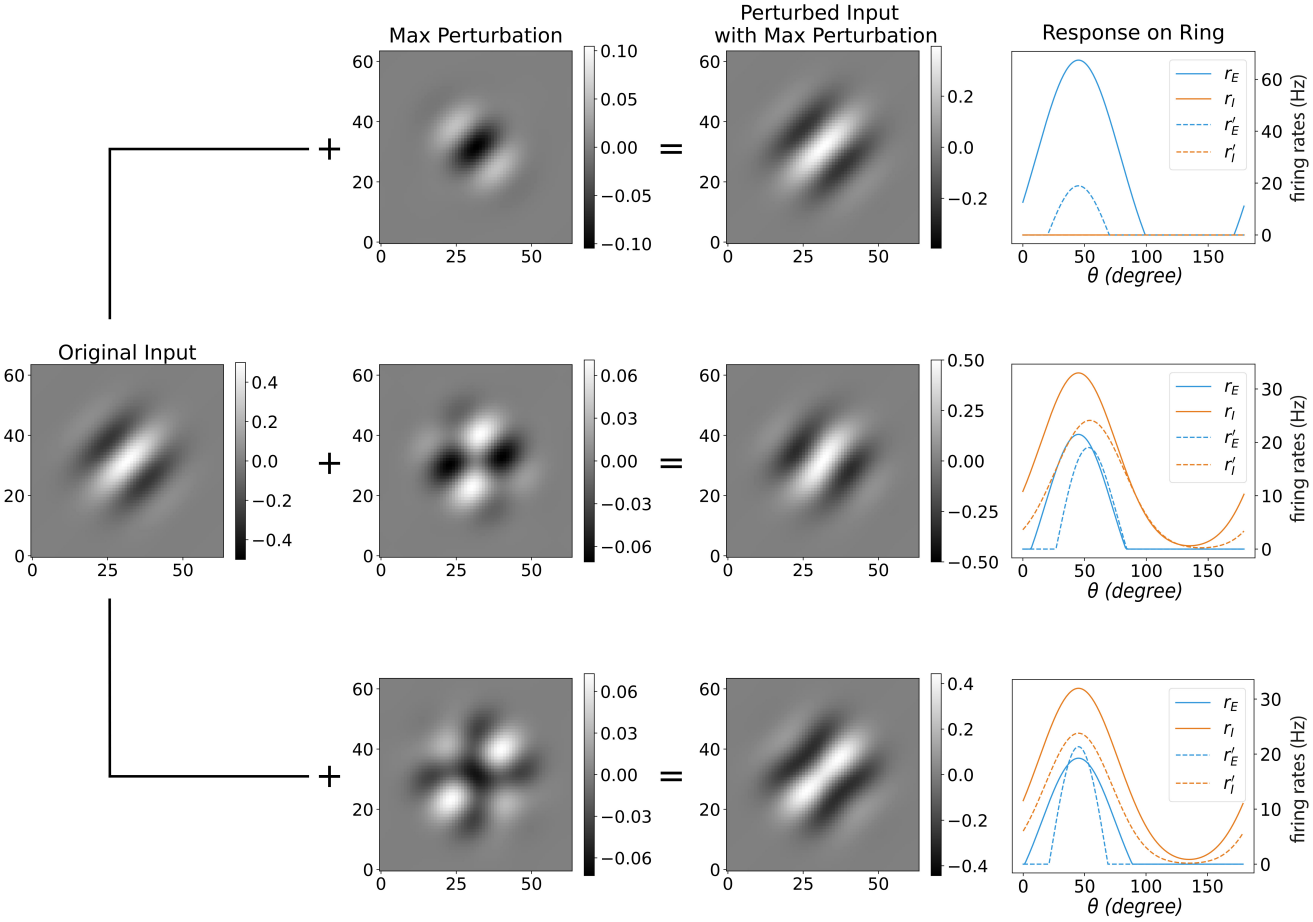
Maximally effective image perturbations for the full pipeline (image $\to F_{G}\to$ ring model). Rows correspond to three lateral–connection regimes around the same base input: **(A)** no lateral connections; **(B)** *g*_*EE*_ = 0.03, *g*_*EI*_ = 0.06, *g*_*IE*_ = 0.04; **(C)** *g*_*EE*_ = 0.01, *g*_*EI*_ = 0.05, *g*_*IE*_ = 0.04. Columns (left → right): original Gabor input; the maximizing image perturbation;the perturbed input; and the ring responses before/after perturbation (excitatory *r*_*E*_ vs. $r_{E}^{\prime }$, inhibitory *r*_*I*_ vs. $r_{I}^{\prime }$; dashed = before, solid = after). The maximizing pattern depends on connectivity: in **(A)** it is *contrast* modulation; in **(B)** it is *rotation*-like; in **(C)** it is *elongation* (envelope aspect ratio). These classes match the orientation–frequency mapping established earlier (contrast ↔ξ = 0, rotation ↔ξ = 1, elongation ↔ξ = 2), showing how frequency-dominant modes translate into image-space perturbations under different lateral profiles.

Next, we analyzed the complete mapping from the image space through the Gabor operator $F_{G}$ into the ring model. For different regimes of lateral connectivity, we identified the image perturbations that maximize the excitatory response under a fixed norm constraint (Figure 7). The maximally effective perturbations fall into three families—contrast, rotation-like, and elongation—consistent with the orientation frequencies ξ = 0, 1, 2 identified earlier.

To quantify these effects, we examined the gain curves (Δ*R* vs. Δ*G*) for three canonical perturbation families: elongation, contrast scaling, and Gaussian noise (Figure 7). The gain curves show that structured perturbations elicit much stronger responses than noise. In regime (A), contrast consistently dominates across amplitudes. In regime (B), elongation initially dominates at small to moderate Δ*G*, but its gain decreases as elongation increases further. Along this path, the maximizing perturbation pattern can shift from elongation-like to rotation-like. This arises because the Gabor filter bank attenuates energy from the center to the periphery; once the base image is highly elongated, further elongation yields diminishing returns, making a small rotation more effective. By contrast, along the contrast and noise paths, elongation remains the strongest perturbation.

The side images illustrate this behavior: the left column shows the input stimulus at different amplitudes, while the right column shows the maximizing perturbation pattern for that input. Elongation corresponds to the stretching of the Gabor envelope, in contrast to global intensity modulation, and noise remains ineffective. The observed preferences are not fixed; they can be systematically altered by tuning the lateral connectivity, as summarized in the phase diagram (Figure 2). In principle, these behaviors are encoded in the frequency response function ĥ_−1_, but here we highlight their manifestation empirically through gain curves.

**Figure 8:**
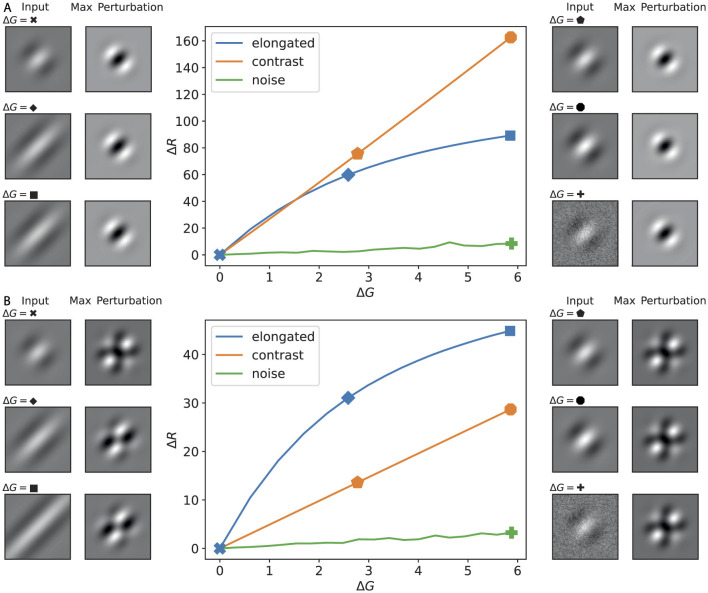
Response functions (Δ*R* vs. Δ*G*) for canonical image perturbations, with examples at selected amplitudes. **(A)** Disconnected lateral connections; **(B)** fully connected regime (cf. Figure 2). Center plots show the change in ring response Δ*R* versus input amplitude Δ*G* for three perturbation families: elongation (blue), contrast scaling (orange), and Gaussian noise (green). Side thumbnails illustrate, for selected amplitudes (indicated by symbols on the curves), the input image **(left)** and the maximizing perturbation pattern for that input **(right)**.

To further compare the impacts of elongation and contrast change, we plot the gain curves in Figure 8. The effectiveness of a perturbation can be assessed from the slope of its curve. It is evident that both patterns produce a larger response than the noise. Furthermore, when we apply all types of lateral connections, as commonly assumed in ring model studies to reproduce orientation selectivity and contrast invariance, the elongation perturbation exerts a stronger influence on the output. In contrast, in the absence of such lateral connections, the contrast change perturbation is more effective.

## 3 Discussion

### 3.1 Advantages and limitations

This study provides insights into the robustness mechanisms of the visual system using the ring model, which represents a simplified network of orientation-selective neurons in the primary visual cortex (V1). One of the key findings is that the system's response to perturbations is strongly shaped by the state of neuronal activation and the pattern of lateral connectivity, which together act as the network's internal priors. Using both a steady-state rate model and a spiking network implementation, we bridge theoretical analysis with biological plausibility.

Our results reveal that distinct activation patterns lead to qualitatively different filtering properties, suggesting that the visual system may adaptively respond to perturbations depending on its current state. This phenomenon echoes the adaptive nature of biological organisms, which continuously adjust to environmental variability (DiCarlo et al., 2012). Moreover, frequency response analysis shows that the strength and spatial structure of lateral connections dictate the degree of selectivity in filtering, supporting the view that biological networks are fine-tuned to enhance relevant inputs while suppressing noise (Carandini and Heeger, 2012). Such findings may inspire more adaptive artificial neural networks.

While omitting inhibitory–inhibitory (I–I) connections and adopting a ReLU activation function simplifies the model, these choices maintain analytical tractability and interpretability. Including I–I connections would mainly shift quantitative gain profiles leftward without altering the core frequency-selective, state-dependent filtering mechanism. Concerning the activation function, we note that softplus can be viewed as a two-segment approximation of ReLU—with slopes 0 and 1, and only a narrow, smooth transition—so our results naturally extend to the softplus case. Similarly, the sigmoid activation introduces an additional saturating segment; however, within the regime of interest in our study (inputs near the threshold), saturation rarely occurs, and the network operates in the quasi-linear region equivalent to ReLU or softplus. Therefore, the qualitative mechanism of state-dependent filtering remains unchanged across these monotonic rectifying nonlinearities.

Despite these advantages, the study also has several limitations. The analytical tractability of the ring model comes at the cost of biological completeness: complex cell-type heterogeneity, long-range feedback, and top-down modulation are not represented, which may restrict the generalization of our findings (Somers et al., 1995).

Although this study does not include a direct comparison between the 1-D ring model and 2-D topology, we postulate that a more realistic local connectivity in a cortical sheet will not make a large difference in the basic mechanism of state-dependent filtering. As other aspects of the two topologies have been studied extensively, there is no qualitative difference. Among them, the notable recent one is the extensive study on response normalization across orientation preferences, contextual (surround suppression), and contrast modulation studies (Ben-Yishai et al., 1995; Somers et al., 1995; Bressloff and Cowan, 2002; Rubin et al., 2015). In our case, the corresponding mechanism for state-dependent filtering can be simply extended to 2-D, but with a caveat of some difficulty in dealing with neurons at the pinwheel center for an ordered orientation map in non-rodents. Specifically, their state-dependence could be very different, and future studies that do employ such geometry can help elucidate such differences.

The present framework can also be interpreted from an adversarial perspective. By locally linearizing the network around a fixed activation state, the system becomes a linear operator represented by the Jacobian *DF*(**r**_0_). Within this local approximation, identifying perturbations of fixed norm that induce the largest change in response is mathematically analogous to constructing adversarial perturbations in machine learning. The leading singular vectors of *DF*(**r**_0_), corresponding to the largest singular values, define a small number of orthogonal directions along which the system is most sensitive. These directions represent the principal modes of effective perturbations—comparable to the dominant adversarial directions in artificial networks. In contrast to conventional adversarial analysis, which exploits such directions to reveal model vulnerabilities, the present study uses them to examine how biological circuits selectively suppress or amplify structured perturbations through state-dependent filtering.

### 3.2 Role of lateral connection to visual robustness

Our analysis reveals that lateral connectivity patterns play a crucial role in determining how the ring model filters out perturbations to an input. Specifically, we find that, with a biologically realistic lateral connection, the network exhibits a stronger response to the elongation of a standard Gabor input compared to changes in contrast. This behavior, which extends beyond linear receptive field modeling, contributes significantly to the system's robustness by prioritizing changes in features relevant to the input over irrelevant signals, such as very low or high-frequency spatial variations or perturbations in the orthogonal orientation.

Furthermore, since lateral connections are shaped by correlations in input statistics, we hypothesize that they selectively prioritize perturbations that align with the input pattern and natural input statistics while filtering out those that deviate from them. This mechanism enhances the robustness of visual information extraction and processing. Given that lateral connectivity is a canonical feature of cortical circuits across layers and brain regions, we propose that it serves as a potential mechanism for filtering out irrelevant perturbations while amplifying relevant ones in response to each input. This results in a system that exhibits remarkable resilience to atypical external disturbances, including adversarial inputs, even in the absence of adversarial training.

Building on this interpretation, our analysis suggests that similar principles could inform architectural designs in artificial networks. For example, lateral recurrent modules with input-dependent gating could emulate state-dependent filtering within convolutional or transformer-based architectures. Such modules would allow feature selectivity to adapt dynamically to input statistics, thereby improving robustness against structured perturbations.

While the ring model simplifies V1 by omitting feedback from higher cortical areas and other hierarchical processing stages (possibly mediated by disinhibition from other inhibitory neurons), it retains the essential feature of lateral connectivity, which serves as the primary mechanism underlying state-dependent filtering. We hypothesize that this lateral connectivity alone, even without complex feedback structures, is sufficient to induce the observed robustness phenomena. The presence of lateral connections creates a system in which perturbations aligned with the network's current state are selectively amplified and irrelevant noise is suppressed, providing a basic form of robustness. This simplified model does not preclude the role of higher-level feedback, but we posit that lateral connectivity is the critical factor driving the robustness observed in our analysis.

In contrast, most current deep-learning architectures—except for transformers—lack mechanisms that incorporate lateral computations analogous to those in biological visual pathways. As a result, these models remain highly vulnerable to small, unseen adversarial perturbations, highlighting a significant gap in robustness between biological and artificial systems.

### 3.3 Future research

Moving forward, several avenues of investigation could further enhance our understanding and application of robustness in visual systems:

1. Hierarchical models: Extending the analysis to hierarchical models of visual processing, including multiple layers that mimic the entire visual pathway, would provide a more comprehensive understanding of robustness across different stages of visual processing (Felleman and Van Essen, 1991).
2. Comparative studies: Conducting comparative studies between ring models and networks without lateral connections will elucidate the advantages of this connectivity in filtering out abnormal patterns and noise (Rubin et al., 2015).
3. Enhanced biological models: Incorporating more detailed biological data into the spiking network models could improve their realism. This could involve complex neurotransmitter dynamics, dendritic processing, and more accurate replication of neural circuitry (Said and Heeger, 2013).
4. Artificial neural networks optimization: Applying insights from biological systems to optimize artificial neural networks could lead to more robust machine learning algorithms. The focus should be on minimizing the sample sizes required for training and improving the network's resilience to input perturbations (Goodfellow et al., 2016).

In summary, this study provides novel insights into the role of state-dependent filtering in shaping robust visual processing. We demonstrate how activation states and lateral connectivity influence neural responses to infinitesimal perturbations and suggest that integrating state-dependent priors into artificial models may improve their adaptability and resilience in complex environments. This perspective bridges the gap between biological and artificial neural networks, offering new directions for both neuroscience and AI research.

## 4 Materials and methods

### 4.1 Steady-state ring model

We use the steady-state rate ring model, which can be viewed as the solution to the steady state of a dynamic system.


$$\begin{array}{l} r_{E}=g\left(I_{E}+k_{EE}*r_{E}-k_{EI}*r_{I}\right),\\ r_{I}=g\left(I_{I}+k_{IE}*r_{E}-k_{II}*r_{I}\right). \end{array}$$ (9)

*r*_*E*_ (*r*_*I*_) is a vector denoting the firing rates of the excitatory (inhibitory) neuron population. *I*_*E*_ (*I*_*I*_) represents the excitatory (inhibitory) external input, and *k*_*XY*_ (*X, Y* ∈ {*E, I*}) is the connectivity kernel from population *Y* to *X*, implementing the Gaussian profile.

The activation function *g* is chosen as the rectified linear unit (ReLU), and * denotes the circular convolution operation, enforcing the ring topology. The mathematical formula is as follows:


$$\begin{array}{l} x*h=\overset{N-1}{\underset{k=0}{\sum }}x\left[k\right]h\left[\left(n-k\right)\;\text{mod }N\right], \end{array}$$ (10)

*x*[*n*] and *h*[*n*] here are of length *N*.

It should be noted that *k*_*XY*_ has a Gaussian profile, which is symmetric about θ = 0. The depiction of *k*_*XY*_ shows a Gaussian profile over the angle range from −90 degrees to 90 degrees. However, when performing the convolution, *k*_*XY*_ should be adjusted from 0 to −180 in the inverse direction. Therefore, the *k* here needs to be shifted. There is some symbol abuse here, and the meaning should be interpreted in context.

With small perturbations δ*I*_*E*_ and δ*I*_*I*_, we obtain the perturbed system:


$$\begin{array}{l} \delta r_{E}=g_{E}^{\prime }⊙\left(\delta I_{E}+k_{EE}*\delta r_{E}-k_{EI}*\delta r_{I}\right),\\ \delta r_{I}=g_{I}^{\prime }⊙\left(\delta I_{I}+k_{IE}*\delta r_{E}\right), \end{array}$$ (11)

⊙ stands for the Hadamard (element-wise) product. Here, *g* is the ReLU function, and each element of *g*′ takes the value 0 or 1, depending on whether the corresponding neuron is active. For convenience, we encode the information of $g_{X}^{\prime }$ in a matrix *G*_*X*_ and derive the matrix-vector form of the perturbed system.


$$\begin{array}{l} \delta r_{E}=G_{E}\left(\delta I_{E}+K_{EE}\delta r_{E}-K_{EI}\delta r_{I}\right),\\ \delta r_{I}=G_{I}\left(\delta I_{I}+K_{IE}\delta r_{E}-K_{II}\delta r_{I}\right), \end{array}$$ (12)

*K*_*XY*_ is a circulant matrix of the lateral connection *k*_*XY*_, with the first row taken from *k*_*XY*_, discretized from angle 0 to −180. Since the kernel *k*_*XY*_ is symmetric about 0, the generated matrix is symmetric.

For clarity, we provide explicit definitions of the matrices *G*_*X*_ and *K*_*XY*_ used in Equation 11.

The matrix $G_{X}\in {\mathbb{R}}^{N\times N}$ is a diagonal matrix encoding the derivative of the activation function for each neuron in population *X* ∈ {*E, I*}. For ReLU activation, it is defined as follows:


$${\left(G_{X}\right)}_{ij}=\;\{\begin{array}{ll} 1, & \text{if }i=j\text{ and }{\left(r_{X}\right)}_{i}>0\\ 0, & \text{otherwise} \end{array}\text{or equivalently, }G_{X}=\mathrm{diag}({g^{\prime }}_{X}),$$ (13)

where $g_{X}^{\prime }\in {\mathbb{R}}^{N}$ is a binary vector with ${\left(g_{X}^{\prime }\right)}_{i}=1$ if the *i*-th neuron is active, and 0 otherwise.

The matrix $K_{XY}\in {\mathbb{R}}^{N\times N}$ is a circulant matrix generated from the lateral connectivity kernel *k*_*XY*_. The first row of *K*_*XY*_ is obtained by discretizing the kernel as follows:


$$\begin{array}{l} {\left(K_{XY}\right)}_{1j}=k_{XY}\left(\theta_{j}\right),\;\theta_{j}=-\frac{180}{N}\left(j-1\right),\;j=1,\ldots ,N, \end{array}$$ (14)

and each subsequent row is a right circular shift of the previous one. Due to the even symmetry of *k*_*XY*_, the resulting *K*_*XY*_ is both symmetric and circulant:


$$\begin{array}{l} K_{XY}=\mathrm{circ}\left(k_{XY}\left(\theta_{1}\right),k_{XY}\left(\theta_{2}\right),\ldots ,k_{XY}\left(\theta_{N}\right)\right). \end{array}$$ (15)

The solution to the equation can be given in a formal expression:


$$\begin{array}{l} \delta r_{E}=(I-G_{E}K_{EE}+G_{E}K_{EI}(I+G_{I}K_{II}{)}^{-1}G_{I}K_{IE}{)}^{-1}(G_{E}\delta I_{E}\\ \;-G_{E}K_{EI}(I+G_{I}K_{II}{)}^{-1}G_{I}\delta I_{I}), \end{array}$$ (16)

$$\begin{array}{l} \delta r_{I}={\left(I+G_{I}K_{II}\right)}^{-1}G_{I}\left(\delta I_{I}+K_{IE}\delta r_{E}\right), \end{array}$$ (17)

Replacing ReLU with softplus or sigmoid preserves the qualitative state-dependent filtering while only smoothing out the active-inactive transition, confirming robustness to the choice of activation function.

### 4.2 Frequency response curve for all neurons active

Perform a Fourier transformation on both sides of the equation with respect to neuronal positions on the ring.


$$\begin{array}{l} {\hat{\delta r}}_{E}=\hat{\delta }I_{E}+{\hat{k}}_{EE}⊙{\hat{\delta r}}_{E}-{\hat{k}}_{EI}⊙{\hat{\delta r}}_{I},\\ {\hat{\delta r}}_{I}={\hat{\delta I}}_{I}+{\hat{k}}_{IE}⊙{\hat{\delta r}}_{E}, \end{array}$$ (18)

Here, the Fourier transformation is in the form


$$\begin{array}{l} \hat{x}=\overset{N-1}{\underset{n=0}{\sum }}x\left[n\right]\exp\left(-ik\omega_{0}n\right),\omega_{0}=\frac{2\pi }{N}. \end{array}$$ (19)

The perturbed system can be solved now directly in the frequency space, and the solution is


$$\begin{array}{l} {\hat{\delta r}}_{E}=\left({\hat{\delta I}}_{E}-{\hat{k}}_{EI}⊙{\hat{\delta I}}_{I}\right){\left(1-{\hat{k}}_{EE}+{\hat{k}}_{EI}⊙{\hat{k}}_{IE}\right)}^{-1},\\ {\hat{\delta r}}_{I}=\left({\hat{k}}_{IE}⊙{\hat{\delta I}}_{E}+{\hat{\delta I}}_{I}-{\hat{k}}_{EE}⊙{\hat{\delta I}}_{I}\right){\left(1-{\hat{k}}_{EE}+{\hat{k}}_{EI}⊙{\hat{k}}_{IE}\right)}^{-1}. \end{array}$$ (20)

The singular values are given by ${\left(1-{\hat{k}}_{EE}+{\hat{k}}_{EI}⊙{\hat{k}}_{IE}\right)}^{-1}$, with the frequency order. As we have mentioned before, the singular values against frequency can be classified into several cases. Here, we give the specific classification basis when the kernels are given as follows:


$$\begin{array}{l} k_{XY}=\alpha_{XY}e^{-x^{2}/2\sigma_{XY}^{2}}, \end{array}$$ (21)

*X, Y* ∈ {*E, I*}. With these Gaussian kernels, we have them in frequency space as follows:


$$\begin{array}{l} {\hat{k}}_{XY}\left(\xi \right)=\frac{N}{T}\alpha_{XY}\sqrt{2\pi }\sigma_{XY}e^{-2\pi^{2}\sigma_{XY}^{2}\xi^{2}/T^{2}}, \end{array}$$ (22)

where *N* is the number of neurons, and *T* is the period, which takes 180 here.

Denote ${\tilde{\alpha }}_{XY}=\frac{N}{T}\alpha_{XY}$, we care about the change of eigenvalues related to the kernel's parameters. We have


$$\begin{array}{l} {ĥ}_{0}=1-{\hat{k}}_{EE}+{\hat{k}}_{EI}{\hat{k}}_{IE}\\ \;=1-{\tilde{\alpha }}_{EE}\sqrt{2\pi }\sigma_{EE}e^{-2\pi^{2}\sigma_{EE}^{2}\xi^{2}/T^{2}}\\ \;+2\pi {\tilde{\alpha }}_{EI}{\tilde{\alpha }}_{IE}\sigma_{EI}\sigma_{IE}e^{-2\pi^{2}\left(\sigma_{EI}^{2}+\sigma_{IE}^{2}\right)\xi^{2}/T^{2}}, \end{array}$$ (23)

There are three key quantities here.

- $\left(\sigma_{EI}^{2}+\sigma_{IE}^{2}\right)/\sigma_{EE}^{2}$. We can view $\sigma_{EI}^{2}+\sigma_{IE}^{2}$ as the scope for the recurrent inhibitory lateral connection and σ_*EE*_ for the excitatory one. Thus, this quantity determines which part has a wider scope.
- ${\tilde{\alpha }}_{EE}\sigma_{EE}/({\tilde{\alpha }}_{EI}{\tilde{\alpha }}_{IE}\sigma_{EI}\sigma_{IE}$ determines whether all the frequencies are uniformly enhanced or suppressed.
- $\left({\tilde{\alpha }}_{EE}\sigma_{EE}^{3}\right)/\left({\tilde{\alpha }}_{EI}{\tilde{\alpha }}_{IE}\sigma_{EI}\sigma_{IE}\left(\sigma_{IE}^{2}+\sigma_{EI}^{2}\right)\right)$ determines whether the zero frequency is enhanced/suppressed the most.

It appears that a condition is missing here. Additionally, more illustrative diagrams could be added to facilitate a thorough discussion of different scenarios. Moreover, references to this section should be incorporated into the earlier content.

### 4.3 Frequency response curve for part of neurons active

Perform a Fourier transformation on both sides of the equation with respect to neuronal positions on the ring


$$\begin{array}{l} {\hat{\delta r}}_{E}=\hat{g_{E}^{\prime }}*\left(\hat{\delta }I_{E}+{\hat{k}}_{EE}⊙{\hat{\delta r}}_{E}-{\hat{k}}_{EI}⊙{\hat{\delta r}}_{I}\right),\\ {\hat{\delta r}}_{I}=\hat{g_{I}^{\prime }}*\left({\hat{\delta I}}_{I}+{\hat{k}}_{IE}⊙{\hat{\delta r}}_{E}\right), \end{array}$$ (24)

### 4.4 Gabor filters

We are interested in how the model is sensitive to changes in images and in the gap between a signal and an image, or vice versa. We bridge the gap with Gabor filters. A Gabor filter is constructed as


$$\begin{array}{l} F_{G}=\left[\begin{array}{c} g_{1}\\ g_{2}\\ \vdots \\ g_{n} \end{array}\right], \end{array}$$ (25)

where $g_{i}=g^{\left(i-1\right)\omega },i=1,2\cdots n$, (ω = 2π/*n*) are row vectors, generated by flattening discretized 2-d Gabor functions with the expression


$$\begin{array}{l} g^{\theta }=g(x,y;A,\text{λ},\theta ,\psi ,\sigma ,\gamma )\\ \;=A\exp(-\frac{{x^{\prime }}^{2}+\gamma^{2}{y^{\prime }}^{2}}{2\sigma^{2}})\cos(2\pi fx^{\prime }+\psi ), \end{array}$$ (26)

where


$$\begin{array}{l} x^{\prime }=x\cos\left(\theta \right)+y\sin\left(\theta \right),\\ y^{\prime }=-x\sin\left(\theta \right)+y\cos\left(\theta \right). \end{array}$$ (27)

### 4.5 Steady-state ring model for given input

In the above discussion, we focused mainly on perturbations; that is, we did not consider the inputs. In this section, we will describe how to extend the previous steady-state ring model to create a mapping from image input to input. We used the following model:


$$\begin{array}{l} I_{E}=F_{G}x_{\text{image}},\\ r_{E}=g\left(I_{E}+k_{EE}*r_{E}-k_{EI}*r_{I}+b_{E}\right)\\ r_{I}=g\left(k_{IE}*r_{E}\right) \end{array}$$ (28)

where $F_{G}$ is the Gabor filter and *b*_*E*_ is the bias of the model. Here, we use a model with added bias, compared to the model from the theoretical analysis. However, this change does not affect the theoretical analysis, as it is based on the amount of variation; the fixed constant is eliminated after the actual input. At the same time, the bias values are hyperparameters that control how many neurons can be activated by an input. This is similar to the fact in biology that neurons are not activated by tiny stimuli, but only when the stimulus reaches a certain intensity.

For an input, we use numerical methods to solve the above equations. After completing the solution, we can obtain the neuron's activation, and with the fixed activation, the perturbation analysis can be performed as described before.

### 4.6 I&F neuronal ring model

In this study, we consider a fully connected network with conductance-based, integrate-and-fire neuron (Obeid and Miller, 2021). The population consists of *N* = 600 neurons, each labeled by its orientation preference, θ_*k*_ = 0.3*k* degrees, which forms a ring. The dynamics of each neuron in the network are modeled by the leaky integrate-and-fire equation:


$$\begin{array}{l} \tau_{m}\frac{\text{d}V}{\text{d}t}=-\left(V-R_{L}\right)+\frac{g_{E}}{g_{L}}\left(R_{E}-V\right)+\frac{g_{I}}{g_{L}}\left(R_{I}-V\right), \end{array}$$ (29)

where τ_*m*_ denotes the time constant, *g*_*L*_ is the leak conductance, *g*_*E*_ and *g*_*I*_ are the time-dependent excitatory and inhibitory conductances, and *R*_*L*_, *R*_*E*_, *R*_*I*_ are reversal potentials. When the neuron's membrane potential reaches threshold *V*_*th*_, the neuron generates a spike, and the membrane potential returns to the resting potential*V*_rest_ and remains at the resting potential until the end of refractory period τ_*ref*_. Biophysical parameters are used: *g*_*L*_ = 10*nS, R*_*L*_ = −70*mV, R*_*E*_ = 0*mV, R*_*I*_ = −80*mV, V*_*th*_ = −50*mV, V*_rest_ = −56*mV*, τ_*m*_ = 15*ms*, τ_*ref*_ = 0*ms*. For any neuron *n* of type *X* ∈ {*E, I*}, *g*_*E*_, *g*_*I*_≥0 are its excitatory and inhibitory conductances governed by


$$\begin{array}{l} \frac{\text{d}g_{E}}{\text{d}t}=-g_{E}/\tau_{E}+\overset{N_{E}}{\underset{b=1}{\sum }}\underset{j}{\sum }W_{XE}\delta \left(t-t_{Ej}\right)+\underset{k}{\sum }g_{X,\text{ext}}\delta \left(t-t_{\text{ext},k}\right), \end{array}$$ (30)

$$\begin{array}{l} \frac{\text{d}g_{I}}{\text{d}t}=-g_{I}/\tau_{I}+\overset{N_{I}}{\underset{b=1}{\sum }}\underset{j}{\sum }W_{XI}\delta \left(t-t_{Ij}\right), \end{array}$$ (31)

where τ_*E*_ = 3ms and τ_*I*_ = 3ms are decay rates for excitatory and inhibitory conductances, respectively. And *W*_*XY*_ = w*g_*XY*_, where $w=A+B*\exp\left(-{\left(\theta_{\text{pre}}-\theta_{\text{post}}\right)}^{2}/\left(2\sigma_{ori}^{2}\right)\right)$, and θ_pre_ is the reference angle for pre-synaptic neurons, θ_post_ is the reference angle for post-synaptic neurons, σ_*ori*_is the width of the Gaussian kernel, and *A, B* are constants satisfying *A*+*B* = 1. Synaptic inputs from other neurons within the network are described in the second terms on the right sides of Equations 29, 30 *t*_*Ej*_, *t*_*Ij*_are the spike times of all the E- and I-neurons pre-synaptic to neuron n. And external synaptic input is described by a Poisson sequence with rates *r*_ext_ that arrives at neuron n in *t*_ext, *k*_. And δ(·) is the Dirac delta function indicating an instantaneous jump of conductance *g*_*E*_ or *g*_*I*_ upon the arrival of an E- or I- or external spike, with amplitude equal to *g*_*E*_, *g*_*I*_, *g*_ext_ respectively. In addition, our Poisson groups and E- and I-neurons are all one-to-one connected (i.e., we have 2*N* Poisson neurons), and the rates of the Poisson groups of neurons with corresponding preferential angles satisfy the following equation:


$$\begin{array}{l} r_{\text{ext}}=A*cos\left(2\pi \theta f\right)+C, \end{array}$$ (32)

where *A* represents the amplitude of the fluctuation, *f* represents the frequency of the fluctuation, θ is the preference angle of the neuron, and *C* is the strength of the base frequency inputs. This equation describes how the input changes spatially, i.e., we can generate stimuli of different spatial frequencies. At the same time, we ensure that the input mean is constant and its variance can be adjusted, i.e., the product of *r*_ext_ and *g*_ext_ should be constant.

If we consider the mean-driven regime mentioned above, we need:


$$\begin{array}{l} N\to +\infty ,\;g_{XY}\to 0,\;N_{input}\to +\infty , \end{array}$$

which means we tune the network primarily based on the mean of the inputs rather than their fluctuations, i.e., the input variance tends to 0. With these parameters, the network will experience even less randomness, reducing fluctuations and bringing it closer to our theoretical analysis.

**Figure 9:**
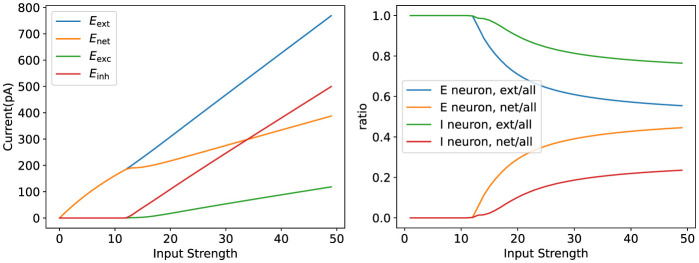
Validation of parameter rationality. The figure on the **left** illustrates the variation of the network current as the input strength varies. The figure on the **right** shows the internal network current versus the external input current as a percentage of the total network current.

Finally, we verified how well the model parameters match biological phenomena, as shown in Figure 9. We demonstrated that the model can reproduce biological observations, such as lateral inhibition and winner-take-all behavior, which are essential for tasks like orientation selectivity.

### 4.7 Measurement of spiking neuron ring model at different input frequencies

Our goal is to test how the ring model's response varies with different frequency inputs under different connectivity conditions (i.e., connection strengths and spatial extents between excitatory and inhibitory neurons). Therefore, we chose to benchmark the ring model's response at different frequencies, without any connections, to test the variation across different connections. Various measurements are accomplished through the following process:

1. Vary the amplitude of the fluctuation *A* when there is no connection and test the change in the issuance rate.
2. At the same time, take the discrete Fourier transform of the change in response and the change in the input current to obtain $\hat{\text{Δ}r}/\hat{\text{Δ}I}$.
3. Change the amplitude of the fluctuation *A* again, but with the corresponding connection, and test the change in the rate of issuance.
4. Take a discrete Fourier transform of the new change in the issuance rate versus the change in input current to get $\hat{\text{Δ}r^{\prime }}/\hat{\text{Δ}I^{\prime }}$.
5. Comparing the two ratios, that is, we end up with $\hat{\text{Δ}r^{\prime }}/\hat{\text{Δ}r}$.

Therefore, in our experiments, we mainly examine the relationship between *A* and $\hat{\text{Δ}r^{\prime }}/\hat{\text{Δ}r}$ as a subject of analysis, which is similar to that analyzed by the steady-state rate model.

### 4.8 Parameter correspondence in mean-driven regime

Since the steady-state rate model is a dimensionless model, we need to consider its parametric correspondence to the actual model with the following equations:


$$\begin{array}{l} r_{E}=g\left(I_{E}+k_{EE}*r_{E}-k_{EI}*r_{I}\right), \end{array}$$ (33)

$$\begin{array}{l} r_{I}=g\left(I_{I}+k_{IE}*r_{E}\right), \end{array}$$ (34)

where $k_{XY}=\alpha_{XY}\exp\left(-x^{2}/\left(2\sigma^{2}\right)\right)$. We fix *g* = 1 and set *r*_*E*_, *r*_*I*_ to match the experimental results in units of Hz. We then calculate *I*_*E*_, *I*_*I*_, α_*XY*_ in the steady-state rate model.

To simplify the description, we denote *I*_*E*_ and *I*_*I*_ as the current of the theoretical model, denote $I_{E}^{\prime }$ and $I_{I}^{\prime }$ as the current of the spiking neuron model, and denote $\bar{r_{E}}$ and $\bar{r_{I}}$ as the mean of all neuron firing rates (only the spiking neuron model needs to be averaged here, and this is due to the randomness it introduces at the time of the experiment). We will complete the correspondence of the parameters by the following process:

1. Determine the correspondence of *I*_*E*_, *I*_*I*_ in the absence of any connection: $r_{E}=I_{E}=kI_{E}^{\prime }+b$.
2. We compute α_*EE*_ when there is only an E-to-E coupling. We consider *r*_*E*_ = *I*_*E*_ + *k*_EE_*r_*E*_, and we have

$$\alpha_{EE}=\frac{\bar{r_{E}}-\left(kI_{E}^{\prime }+b\right)}{\bar{r_{E}}\int_{0}^{180}e^{-x^{2}/\left(2\sigma^{2}\right)}dx}.$$

3. We compute α_*IE*_ when there is only an I to E coupling. We consider *r*_*E*_ = *I*_*E*_ − *k*_IE_*r_*I*_, and we have

$$\alpha_{IE}=\frac{\bar{r_{E}}-\left(kI_{E}^{\prime }+b\right)}{\bar{r_{I}}\int_{0}^{180}e^{-x^{2}/\left(2\sigma^{2}\right)}dx}.$$

4. We compute α_*EI*_ when there is only an E to I coupling. We consider *r*_*I*_ = *I*_*I*_ + *k*_EI_*r_*E*_, and we have

$$\alpha_{EI}=\frac{\bar{r_{I}}-\left(kI_{I}^{\prime }+b\right)}{\bar{r_{E}}\int_{0}^{180}e^{-x^{2}/\left(2\sigma^{2}\right)}dx}.$$

Finally, we can reasonably compare the steady-state rate model with the distribution model.

## Data Availability

The datasets presented in this study can be found in online repositories. The names of the repository/repositories and accession number(s) can be found in the article/Supplementary material.
