# Supplementary material for: State-dependent filtering as a mechanism toward visual robustness
Source: Front Comput Neurosci. 2025 Dec 10;19:1699179. doi: 10.3389/fncom.2025.1699179 (PMC12728028; doi:10.3389/fncom.2025.1699179)
Supplement: Supplementary file 1 [file Data_Sheet_1.pdf]

Appendix

A Gap between steady-state rate models and spiking neuron models

In the analysis of the steady-state rate model, we can see that the model does not enhance or suppress the response to the high-frequency part. This is because $k_{EE} * r_E, k_{EI} * r_I, k_{IE} * r_E$ are constants at high frequencies, that is, they do not have high-frequency components. Based on this observation, we can analyze similar parts of the spiking neuron model $\frac{g_E}{g_L}(R_E - V)$, $\frac{g_I}{g_L}(R_I - V)$, and analyze whether they change in the high-frequency part.

Through experiments, we found that, unlike the steady-state rate model, the potential fluctuates in the spiking neuron model by fluctuating inputs, as shown in Fig 1. We believe that this fluctuation in potential affects the model's performance in the high-frequency part. That is, models with lateral connections will somewhat enhance or weaken the high-frequency part, compared to models without connections.

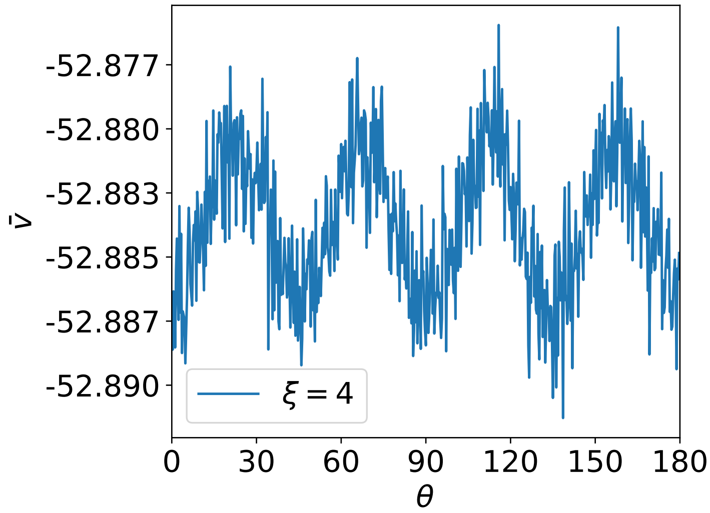

Figure 1: The curve shows the orientation-dependent fluctuation of the average membrane potential $\bar{v}$ across neurons over time for the mode $\xi = 4$. Here, $\bar{v}$ denotes the temporal average of each neuron's membrane potential. The periodic modulation in $\bar{v}$ implies that the potential of a neuron fluctuates as a result of inputs.

Based on the above understanding, we will consider a more simplified model to analyze how fluctuations in potential can have an effect in a model with lateral connections. We consider the equation in the steady state, i.e., we regard $g_E, g_I, g_{ext}, g_{in}$ (with spatial fluctuations) as constants for analysis, and we can get the following equation:

$$\frac{\mathrm{d}v}{\mathrm{d}t} = -a(v - \frac{b}{a}). \tag{1}$$

This is a simplification of the above equation. At the same time, we consider the average potential during the two firing processes, and even more simply, we consider the average potential $\bar{v}$ from $t = 0, v = v_0$ to the first time the firing potential is reached $t = t_1, v = v_1$ (this moment is related to a and b), we get the following result:

$$\bar{v} = \frac{b}{a} + \frac{v_0 - v_1}{\ln \frac{av_0 - b}{av_1 - b}} \tag{2}$$

$$t_1 = \frac{1}{a} \ln \frac{av_0 - b}{av_1 - b} \tag{3}$$

That is, $\bar{v}$ will vary with the connection parameters and the firing rate, where t_1 is affected by the firing rate. On this basis, we give the values of a and b :

$$a = \frac{g_L + g_{\text{ext}} + g_E + g_I}{\tau_m g_L}, b = \frac{R_L g_L + R_E(g_{\text{ext}} + g_E) + R_I g_I}{\tau_m g_L}. \tag{4}$$

Then, we consider the input as:

$$g_{\text{ext}} = C + \text{amp} * \cos(2\pi\theta * \text{fre}). \tag{5}$$

Under the condition of fixed input strength and fluctuation strength, we found that there is inconsistency in the spatial potential when increasing the values of g_E, g_I as shown in Fig. 2.

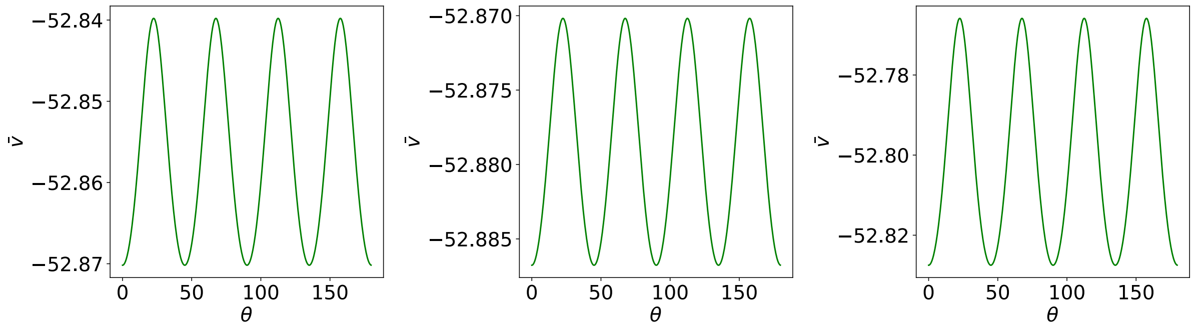

Figure 2: Theory result of average membrane potential, $\bar{v}$: (left) Benchmark: C=12, amp=1.5, $g_E = g_I = 0$, (middle) Increase E connection: $g_E = 3$, (right) Increase I connection: $g_I = 3$.

We can find that the impact of the fluctuation amplitude is inconsistent, and more specifically, we analyzed this inconsistency as shown in Fig 3. The negative part represents the increase in g_E, and the positive part represents the increase in g_I. Based on observations, we can find that the impact of the model on g_E, g_I is inconsistent, that is, it should be a nonlinear change rather than a linear change. That means that, at the same strength, the I connection naturally brings more fluctuation changes than the E connection in the high-frequency part.

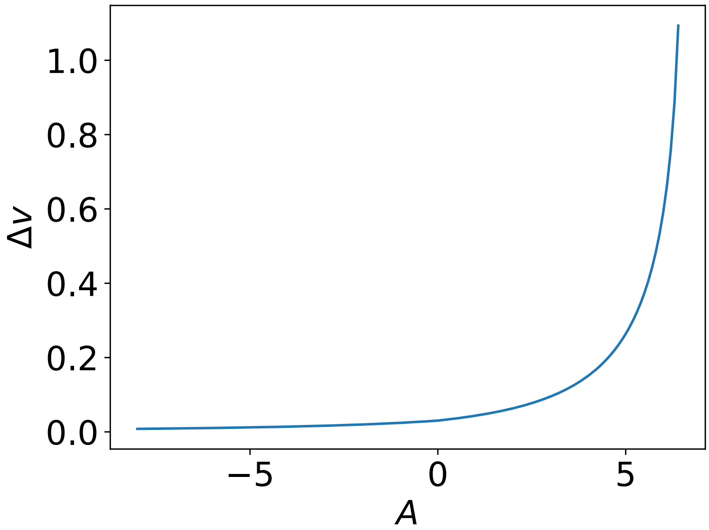

Figure 3: Effect of different connections on the range of fluctuations. A represents the amplitude of g_E and g_I, where a negative number indicates the strength of g_E and a positive number indicates the strength of g_I. This implies that inhibitory connections lead to stronger fluctuations than excitatory connections.

B Fluctuation-driven regime

We first present the key parameters adopted under the mean-driven regime. Unless specifically mentioned, they are consistent with the parameters selected earlier in the text. Here, we set the number of neurons $N = 240$, $N_{\text{input}} = 1$, and the refractory period $\tau_{\text{ref}} = 3ms$ and keep the other parameters the same. The settings for the fluctuation-driven regime will introduce more noise into our model and bring

about greater nonlinearity. We will first demonstrate the results under the fluctuation-driven regime and analyze the differences between mean-driven regime and fluctuation-driven regime.

In fluctuation-driven regime, we obtain results that are qualitatively consistent with the theoretical results. As shown in the Figure 4, when no connections were present, the ring model responded uniformly to all frequencies. With only recurrently excitatory connections, the ring model enhanced the low-frequency response. In contrast, when only recurrently inhibitory connections were present, the ring model suppressed the low-frequency response. And when all connections are present, the model demonstrates a choice of specific frequency.

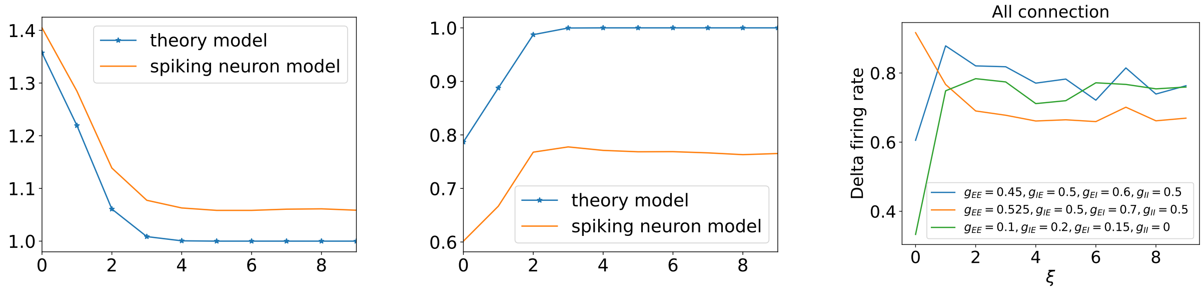

Figure 4: Gain curves of spiking neuron model(fluctuation-driven regime). The figure on the left shows the results with only recurrent excitation connections. The middle figure shows the results with only recurrent inhibition. The right figure demonstrates the results with full connectivity. Due to the complexity of full connectivity, its quantitative comparison with theoretical models is difficult. Therefore, we only show the results of multiple full-connectivity cases that qualitatively match with our theoretical model.

Although these results are qualitatively consistent, quantitatively we can still find differences between fluctuation-driven regime and mean-driven regime. From the response curves, it is evident that the mean-driven regime has almost no fluctuations, whereas the fluctuation-driven rigime exhibits strong fluctuations, as shown in Figure 5. Further, we observe the firing rates in the frequency space, as shown in Figure 6. Compared to the mean-driven regime, the ring model shows a notably enhanced response at the fundamental frequency. In response to the fundamental frequency, analyzing the neuronal population gain curve can help us understand this phenomenon. We have plotted the gain curve of the neuronal population, as shown in Figure 7. We find that in the fluctuation-driven regime, it is more challenging to find an approximately linear region in the neuron's gain curve, unlike in the mean-driven regime. When the gain curve is linear, there is no significant enhancement of the fundamental frequency; however, in the nonlinear region, the response strength at the fundamental frequency increases. These nonlinearities arise not only from the input but also, in part, from the excitatory and inhibitory connections between neurons, which further complicates the quantification of these differences.

Through these analyses, we delve deeper into the complex dynamics of the neuronal network under different driving regime, highlighting the complex balance between network structure, connectivity, and input characteristics. Overall, as long as qualitative consistency is maintained, the key property we propose—state-dependent filtering—remains intact under fluctuation-driven regimes. insights into the adaptability and functionality of neural circuits in varying operational states.

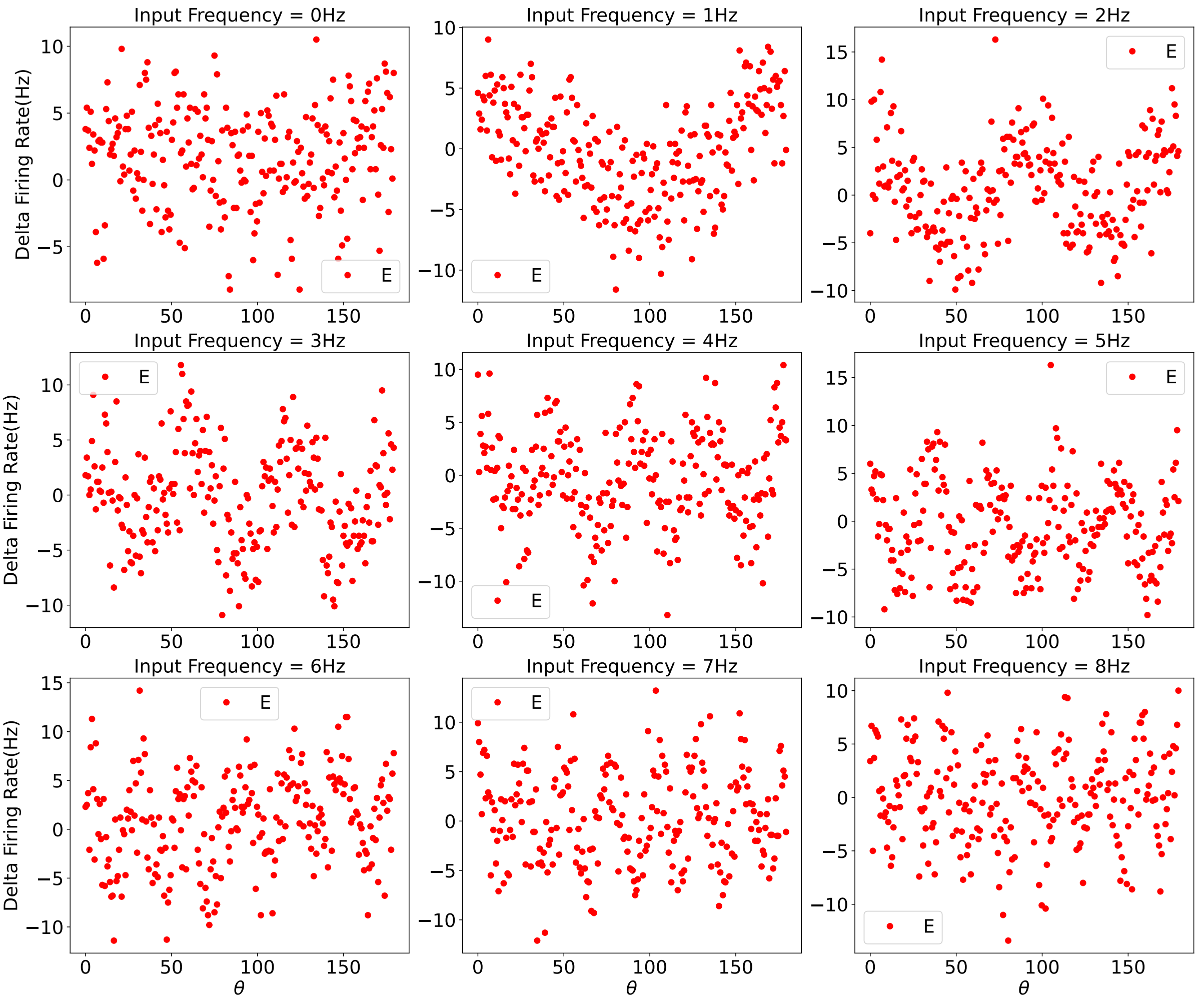

Figure 5: The effect of perturbation on firing rates. This figure shows the change in frequency space brought about by different frequency inputs in the case of full connectivity

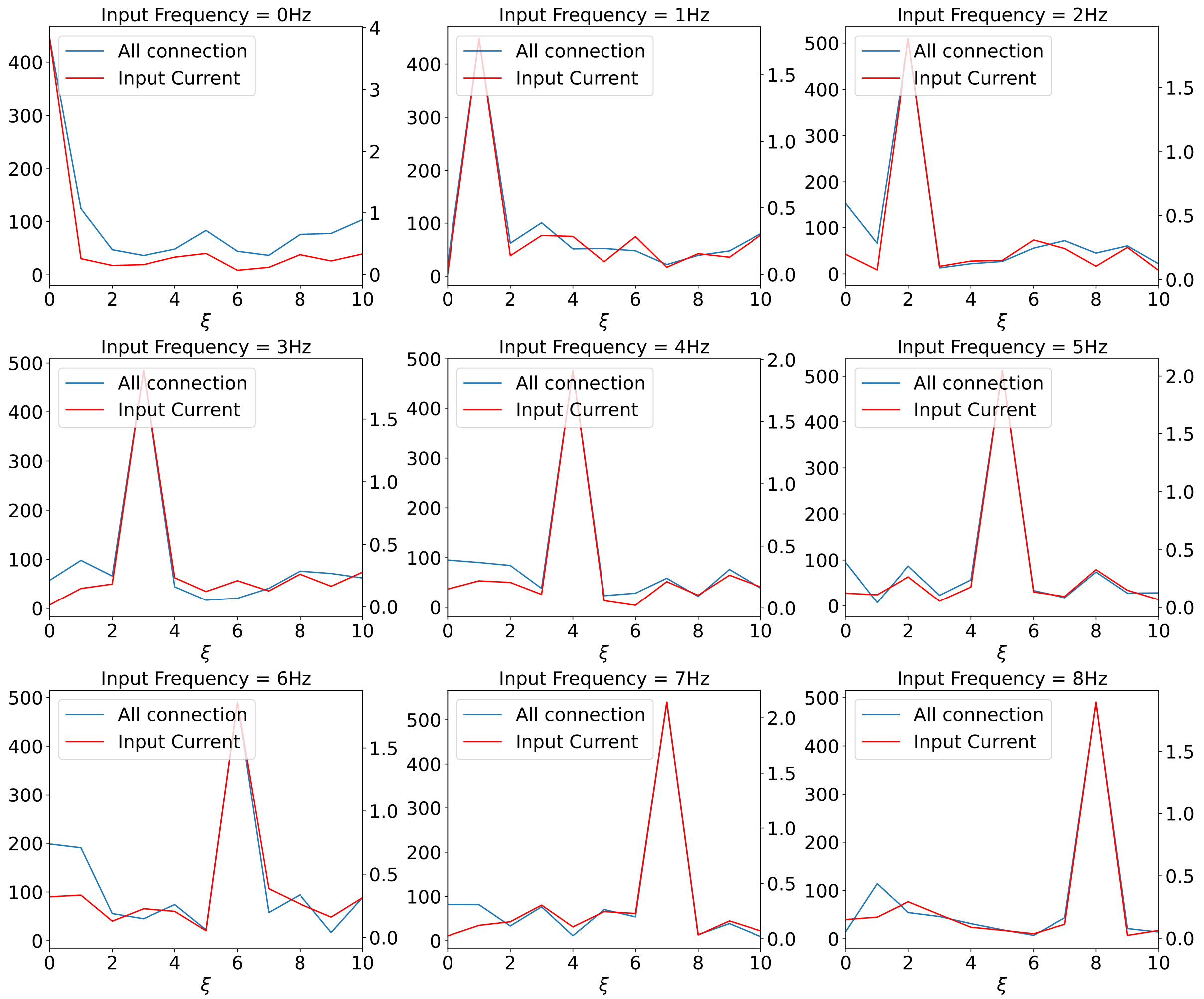

Figure 6: The effect of perturbation on firing rates in frequency space. This figure shows the change in frequency space brought about by different frequency inputs in the case of full connectivity

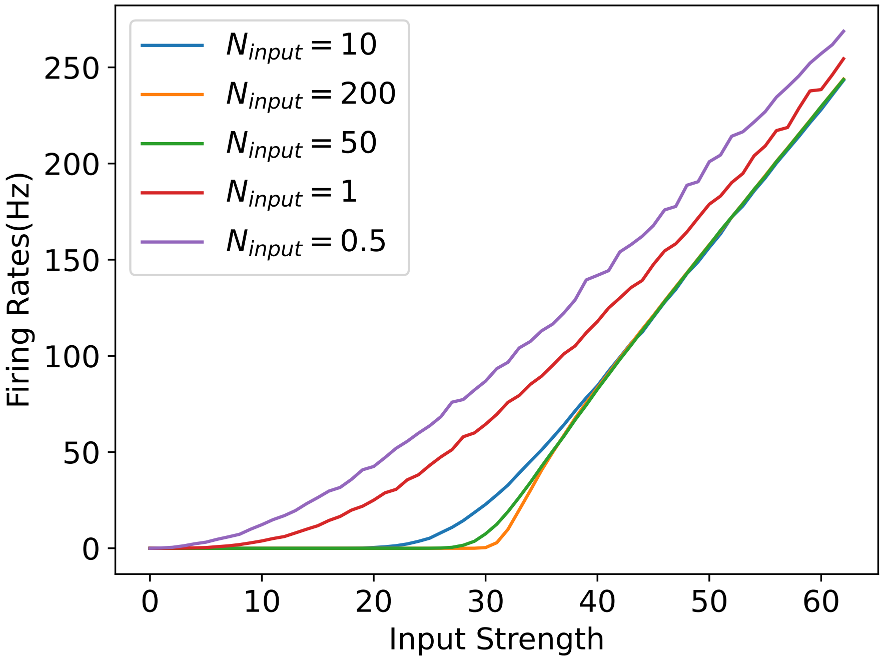

Figure 7: The effect of the variance of the inputs. A smaller N_{input} corresponds to a larger variance.
